# Genome-Based Prediction of Time to Curd Induction in Cauliflower

**DOI:** 10.3389/fpls.2018.00078

**Published:** 2018-02-05

**Authors:** Arne Rosen, Yaser Hasan, William Briggs, Ralf Uptmoor

**Affiliations:** ^1^Faculty of Agriculture and Environmental Science, University of Rostock, Rostock, Germany; ^2^Institute of Horticultural Production Systems, Leibniz Universität Hannover, Hannover, Germany; ^3^Syngenta Seeds B.V., Enkhuizen, Netherlands

**Keywords:** quantitative trait loci, genomic selection, phenology model, cauliflower, vernalization, curd induction

## Abstract

The development of cauliflower (*Brassica oleracea* var. *botrytis*) is highly dependent on temperature due to vernalization requirements, which often causes delay and unevenness in maturity during months with warm temperatures. Integrating quantitative genetic analyses with phenology modeling was suggested to accelerate breeding strategies toward wide-adaptation cauliflower. The present study aims at establishing a genome-based model simulating the development of doubled haploid (DH) cauliflower lines to predict time to curd induction of DH lines not used for model parameterization and test hybrids derived from the bi-parental cross. Leaf appearance rate and the relation between temperature and thermal time to curd induction were examined in greenhouse trials on 180 DH lines at seven temperatures. Quantitative trait loci (QTL) analyses carried out on model parameters revealed ten QTL for leaf appearance rate (LAR), five for the slope and two for the intercept of linear temperature-response functions. Results of the QTL-based phenology model were compared to a genomic selection (GS) model. Model validation was carried out on data comprising four field trials with 72 independent DH lines, 160 hybrids derived from the parameterization set, and 34 hybrids derived from independent lines of the population. The QTL model resulted in a moderately accurate prediction of time to curd induction (*R^2^* = 0.42–0.51) while the GS model generated slightly better results (*R^2^* = 0.52–0.61). Predictions of time to curd induction of test hybrids from independent DH lines were less precise with *R^2^* = 0.40 for the QTL and *R^2^* = 0.48 for the GS model. Implementation of juvenile-to-adult phase transition is proposed for model improvement.

## Introduction

Floral or curd induction is a temperature-mediated process in cauliflower. While temperature optima for curd induction are around 15°C for many summer cultivars, temperatures above 20°C delay or inhibit curd induction. Wide harvest windows resulting from high ambient temperatures lead to several selective harvests on the same field causing high workloads for growers (Wiebe, 1980). Phenology models are useful tools to cope with uncertainties in time to curd induction and harvest (Jensen and Grevsen, 2005). Several modeling approaches have been made to predict harvest time in cauliflower: The earliest models used linear relations between curd diameter and temperature sum to predict time to maturity, which required curd diameter measurements in the field (Salter, 1969; Hand, 1988; Wurr, 1989; Wurr et al., 1990a,b). Later models integrated earlier development stages beginning at transplanting (Pearson et al., 1994; Grevsen and Olesen, 1994). Several models divided development before curd induction into the juvenile phase, ending after a certain number of leaf primordia is initiated and during which plants are insensitive to vernalization, and the adult vegetative or vernalization phase, characterized by temperature sensitivity (Wiebe, 1972a,b,c; Wurr, 1989; Kage and Stützel, 1999). Olesen and Grevsen (2000) established a model simulating time to maturity and enabling curd quality prognosis based on weather data. Jensen and Grevsen (2005) presented a web-based management tool for growers and wholesale traders predicting harvest time by applying the temperature-sum rule. In addition to crop phenology, Kage et al. (2001a,b) modeled the dry matter production and partitioning by means of absorbed photoactive radiation and light use efficiency.

The mentioned models are valid for single or few cultivars. Using crop growth or phenology models in plant breeding was firstly proposed by Yin et al. (2003) via integrating QTL and ecophysiological models. QTL-based phenology models have already been established for crops like wheat (Bogard et al., 2014), barley (Yin et al., 2005b), soy (Messina et al., 2006), and *B. oleracea* (Uptmoor et al., 2012). The studies revealed that QTL can be detected for model parameters describing the response curve of time to flowering, heading or floral induction in relation to the environmental factors photoperiod and/or temperature and that flowering time of progenies within bi-parental crosses can be predicted based on QTL effects. However, most of the studies used no or only few independent lines from the respective populations for model validation, which is apart from QTL detection a prerequisite for possible applications in plant breeding. GS was first introduced in animal breeding (Meuwissen et al., 2001) and has emerged as a new tool in plant breeding by using genome-wide markers and their corresponding effects to predict phenotypes of untested genotypes (Desta and Ortiz, 2014). While most GS studies were conducted in populations with a broad genetic basis, also bi-parental crosses were used (Lorenza and Bernardo, 2009). Combined GS and phenology models have already been tested and are likely to replace QTL-based modeling approaches since predictions can be made across bi-parental populations (Heslot et al., 2014; Technow et al., 2015; Onogi et al., 2016; Uptmoor et al., 2016). Both, QTL-based modeling approaches and combined GS and crop models may dissect complex traits into underlying physiological factors and have in common that QTL or marker effects are estimated for parameters of the response curves of the trait or underlying physiological factor to environmental influences like temperature. While QTL-based modeling approaches consider only main effect loci, GS models estimate effects for all markers and, thus, capture small effect loci as well.

Since *B. oleracea* is closely related to *Arabidopsis thaliana* (Lagercrantz et al., 1996), for which the main regulators of flowering time are known (Jack, 2004; Blázquez, 2005), QTL and flowering-time regulator co-localizations gave hint that these genes may account for flowering-time variation in *B. oleracea*. One important flowering time integrator is *FLOWERING LOCUS C* (*FLC*), an inhibitor of flowering, which is downregulated by vernalization and has a significant impact on flowering-time variation in *A. thaliana* (Koornneef et al., 1994). The *B. oleracea* genome carries several *FLC* homologs (Lagercrantz et al., 1996; Okazaki et al., 2007; Axelsson et al., 2001) and co-localizations with flowering-time QTL indicated that allelic variation of *FLC* might play an important role in flowering-time regulation in *B. oleracea* (Schranz et al., 2002; Brown et al., 2007). However, Okazaki et al. (2007) and Razi et al. (2008) suggested that several tested *FLC* homologs unlikely induce flowering-time variation in *B. oleracea.* While Ridge et al. (2015) found evidence for functional relevance of *BoFLC2* in floral induction, Matschegewski et al. (2015) found no consistent *BoFLC2* transcription patterns in cauliflower breeding lines for different climates and seasons. QTL for curd induction were not only identified in regions harboring *FLC* and *FRI* homologs but also on O6, where *BoAP1-a* and *BoAP1-c* are located (Hasan et al., 2016).

Despite the efforts spent in elucidating genetic mechanisms underlying floral induction in cauliflower, it is still widely unknown, how temperature-dependent development toward curd induction is regulated, which, in turn, hampers the development of new cultivars with stable time to maturity in a broad range of different environments. Identification of candidate genes underlying flowering-time QTL may benefit from integrated modeling approaches since model parameters allow to distinguish vernalization from flowering-time *per se* QTL. To date, most studies integrating genetic and eco-physiological models used QTL rather than genome-wide marker effects, even though state of the art marker-assisted plant breeding strategies rely on GS. Therefore, the present study aims at (1) the identification of QTL for parameters of a phenology model, assuming a linear relation between temperature and thermal-time to curd induction, (2) model parameterization based on both QTL and genome-wide marker effects, (3) model evaluation on multi-environment field data from independent genotypes including DH-lines *per se* and lines crossed with a tester, and (4) comparison of both parameterization principles.

## Materials and Methods

### Plant Material

The present study was carried out on a population consisting of 265 DH lines derived from the F1 of a cross between homozygous parental lines P1 with good harvest-time reliability at higher temperatures and P2 that is less resistant to elevated temperatures but produces high-quality curds. More details about reliability in time to curd induction and harvest time of the PLs are provided by Hasan et al. (2016). A subset of 180 lines was used for model parameterization, while the validation sets included three subsets with 72 additional DH lines *per se*, 160 test hybrids from the parameterization set, and 34 test hybrids obtained from the 72 additional DH lines. Plant material was obtained from SYNGENTA Seeds B.V., Enkhuizen, Netherlands. The PLs were genotyped with a 20k *B. oleracea* Illumina Infinium iSelect single-nucleotide polymorphism (SNP) array (Matschegewski et al., 2015). A subset of SNPs of the array was selected based on polymorphisms between PLs and genome coverage to genotype the DH-population with 176 SNP markers. The genetic map was constructed using MapMaker 3 and the Haldane function. The total map length was 891.2 cM spanning over nine chromosomes. Average distance between SNP markers was 5.3 cM. Physical marker positions are available from Hasan et al. (2016).

### Model Parameterization Trials

Model parameterization is based on controlled temperature treatments described in Hasan et al. (2016). Six trials were carried out in greenhouses with mean temperatures of 11.8, 15.5, 17.3, 19.0, 21.4, and 27.0°C and one in climate chambers with a mean air temperature of 26.5°C. Seeds of all DH lines of the parameterization set were sown in seedling trays and raised in the greenhouse at 22°C until all plants had two to three visible leaves. Afterward, seedlings were planted into 3 l pots, transferred to the temperature treatments in greenhouses or climate chambers, and arranged in randomized complete block designs. Plants were grown at a photoperiod of 16 h, providing 200 μmol m^-2^ s^-1^ additional light by Phillips SON-T Agro lamps when day length was below 16 h or natural radiation during the day below 5 klux. Plants were fertigated daily with 0.5 g l^-1^ Scotts Universal solutions. All trials were terminated after 120 days.

Time to visible curd induction was recorded daily. A curd diameter of 1 cm was used as threshold for considering visible curd induction to have been occurred. Number of leaves larger than 1 cm was counted twice a week. Leaf appearance rate (LAR) was computed as the slope of the regression of leaf numbers on growing degree-days assuming a base temperature of zero.

### Model Validation Trials

For model validation, a field trial was carried out with 72 lines in 2013 in Rostock, Germany. Twelve seeds per line were sown on May 6 into seedling trays and raised in the greenhouse. Eight plants of each line were planted into the field on June 5 when most plants had two to three leaves. Field trials were carried out in a randomized complete block design with two replications and four plants per replication. Seedlings were planted in rows with distances of 50 cm between and within rows. A border row was planted to provide similar conditions for all plants. Irrigation was applied as required and the plot was covered with a net to protect plants during the first weeks from cabbage fly (*Delia radicum*) and other pests. The trial ended on August 16. Time to curd induction was scored twice a week. Beginning of curd formation was defined as curd diameter ≥ 1 cm.

Additional validation trials with similar growing conditions were carried out in 2013 in Ruthe, Germany and in 2011 and 2012 in Zeewolde, Netherlands. In Ruthe, seeds of the validation set were sown on May 14 and eight plants per line were transplanted to the field on June 13 when they had two to three visible leaves. Zeewolde field trials were carried out on the whole DH-population and transplanting took place on June 14, 2011 and May 24, 2012. Mean air temperatures during the experiments were 17.9°C in Rostock, 19.0°C in Ruthe, and 17.4 and 17.2°C in Zeewolde 2011 and 2012, respectively. Only 44 DH lines were used in Zeewolde 2011, whilst all other trials comprised the whole DH *per se* validation set.

Further trials were conducted on the 160 F1 test-hybrids derived from the parameterization set and the 34 hybrids in Ócsa, Hungary, where plants were transplanted on July 7, 2011 and July 6, 2012 and in Zeewolde, where transplanting took place on June 13, 2012. Mean air temperatures in Ócsa were 21.4 and 22°C, respectively; mean air temperature in Zeewolde was 17.4°C. Harvest time was recorded in all Ósca and Zeewolde trials. Sowing was assumed to have taken place 30 days before transplanting and duration from visible curd induction to harvest was assumed to be 30 days (**Table 3**).

### Data Analysis and Model Description

The cauliflower phenology model was divided into a juvenile and a vernalization phase. During the juvenile phase, plants were assumed to be insensitive to temperature, i.e., development followed the temperature-sum rule. Although it is known that genotypic variation in the number of initiated or visible leaves at juvenile-to-adult transition exists (Hand and Atherton, 1987), juvenility was assumed to end for all genotypes after 7 leaves were visible. In contrast, LAR and phyllochron (*P*, d), the reciprocal of LAR, was assumed to be genotype specific. The genotype specific temperature sum for juvenile-to-adult transition (*TS_f_*) was calculated as follows:

$${TS}_{f}=7\times P$$

The model assumed genetic variation in temperature response from end of juvenility to visible curd induction (curd diameter > 1 cm). Accelerating or retarding temperature effects on phenological development may end at floral transition, which, however, was not observed. Instead, the relation between thermal time to visible curd induction in seven different treatments and temperature was estimated by linear regression, where the intercept *T*_0_ (°Cd) describes the extrapolated (theoretical) minimum thermal time to curd induction and the slope *S* (°Cd °C^-1^) the temperature sensitivity, i.e., a genotype with *S* = 0 is insensitive while a genotype with a large positive *S* shows strong vernalization response. The daily development rate *k_ij_* (d^-1^) for the *i^th^* day and *j^th^* line was calculated based on daily mean air temperatures *T_i_* as follows:

$$k_{ij}=\frac{T_{i}}{\left(T_{i}\times S_{j}+T_{0j}\right)}$$

*k_ij_* was added up until the cumulative *k_j_* became ≥ 1, i.e., when the adult vegetative phase was completed and curd development started.

A linkage map was constructed with MapMaker 3 by simultaneous multipoint-analysis using the Haldane function (Lander et al., 1987; Lincoln et al., 1993). On basis of the results of parameterization trials, QTL analyses were conducted on *LAR*, *S*, and *T*_0_ using PlabQTL 1.2 (Utz and Melchinger, 1996). Composite interval mapping (CIM) based on multiple regression with co-factors was applied (Haley and Knott, 1992; Jansen and Stam, 1994; Zeng, 1994; Miller, 2002). The LOD threshold to define a significant QTL was 2.5. The linkage map was drawn with MapChart 2.3 (Voorrips, 2002).

Additive QTL effects were assigned to all detected loci and QTL based parameters for *T*_0_, *S*, and LAR were calculated using the following equation:

$$y_{j}=m+\Sigma e_{i}g_{ij}$$

where *y_j_* is the estimated value of parameter *y* of the *j^th^* line, *m* is the population mean for the parameter, *e_i_* is the additive effect of the *i^th^* QTL, and *g_ij_* is the allelic state of the *i^th^* QTL in line *j*. *g_ij_* can be -1 or +1.

In addition to linkage mapping, marker effects of the three parameters were estimated by genomic selection using the ridge-regression best linear unbiased prediction (rrBLUP) package for R 3.2.4. with the following restricted mixed model (REML) solution:

y=Xβ+Zv+ϵ

where *y* is the vector of phenotypic observations, *X* is the matrix of fixed effects β, *Z* is the matrix of random effects υ, and ε is the residual effects matrix (Endelman, 2011). rrBLUP was carried out on the three model parameters *LAR*, *S*, and *T*_0_. Effects were assigned to all markers and the sum of marker effects was used to calculate additive genomic estimated breeding values (GEBV) of the individuals of the validation sets. GEBVs for *LAR*, *S*, and *T*_0_ were implemented into the phenology model as parameter input values to simulate heading date of the individuals in field trials.

## Results

### Phenotypic Variation for Traits and Model Parameters

Thermal time from transplanting to curd induction under different constant temperatures was measured in the parameterization trials. In general, the higher the temperature, the higher was the temperature sum required for curd induction. Population means ranged from 753 to 1590°Cd (**Table 1**).

**Table 1:** Population means and standard deviations (SD) of temperature sums (TS) required for visible curd induction at different temperatures.

Temperature [°C]	TS parameterization set [°Cd]		TS parental lines [°C]	
		
Mean	Mean	*SD*	P1	P2
11.75	1533	63.6	1629.5	1556.7
15.51	1462	65.7	1640.8	1519.8
17.33	1557	85.6	1923.8	1516.5
19.03	1570	85.4	1868.5	1607.8
21.44	1930	242.3	–	1888.4
26.49	1862	192.5	–	1911.1
27.00	2370	384.2	–	2346.0


**Shown are means across DH-lines used for model parametrization TS of parental lines*.*

Mean LAR of individual genotypes averaged across all parameterization trials ranged from 0.016 to 0.029. The population mean was 0.022 with a standard deviation of ±0.002. Mean *S* was 50.0 (±20.5) and ranged from 4.2 to 106.0. Population mean of *T*_0_ was 631°Cd ± 296°Cd (**Table 2**). Mean *R^2^* of the relation between temperature and thermal time to curd induction was 0.65 (±0.13), maximum *R^2^* was 0.87 and minimum was 0.05.

**Table 2:** Population mean, standard deviation, and maximum and minimum estimates for leaf appearance rate (LAR), slope (S), and intercept (T_0_) of DH lines used for model parameterization and estimates of the parental lines P1 and P2.

Parameter	Mean	*SD*	Max.	Min.	P1	P2
LAR	0.0216	0.0021	0.0293	0.0161	0.0161	0.0214
*S*	50.0	20.5	106.0	4.17	39.8	46.7
*T_0_*	631.3	296.5	1374.6	-194.6	1133.2	840.3


In the validation trials the thermal time to curd induction was measured for the parameterization set, the validation set and F1 hybrids of both sets. Mean thermal time to curd induction ranged from 1276.3 to 1597.7°Cd across all trials and the different subsets (**Table 3**).

**Table 3:** Number of lines of the parameterization and validation sets used in field trials at different locations in 2011, 2012, and 2013; mean, minimum and maximum temperatures during field trials and means with standard deviations (SD), minima and maxima for time to curd induction.

Trial	Zeewolde 2011	Zeewolde 2012	Ruthe 2013	Rostock 2012	Ócsa 2011	Ócsa 2012
**Number of lines**						
Parameterization set (DH lines *per se*)	140	154	–	–	–	–
Validation set (DH lines *per se*)	44	69	68	67	–	–
Parameterization set (F1 hybrids)	–	156	–	–	134	146
Validation set (F1 hybrids)	–	31	–	–	20	24
**Temperature [°C]**						
Mean	17.4	17.2	19.0	17.9	21.4	22.0
Min.	13.0	9.1	12.1	11.7	14.9	13.6
Max.	25.4	21.9	26.9	27.4	29.6	29.4
**Thermal time to curd induction [°Cd]**						
Mean	1276.3	1261.7	1417.0	1494.7	1427.4	1597.7
*SD*	102.6	73.5	139.4	99.5	82.9	135.2
Min.	1075.3	1006.8	1178.2	1237.9	1291.3	1364.0
Max.	1477.2	1509.6	1810.6	1750.4	1621.3	1868.7


### QTL and Genome-Wide Marker Effects for Model Parameters

**FIGURE 1:**
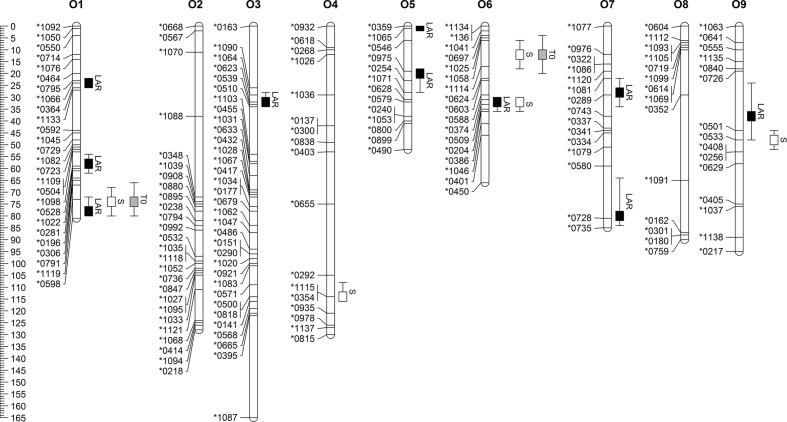
Linkage map of *B. oleracea* with marker names and positions and confidence intervals of QTL for leaf appearance rate (LAR), slope (*S*) and Intercept (*T*_0_).

QTL analyses were carried out on model parameters LAR, *S*, and *T*_0_. In total, ten QTL were detected for LAR. Three of them were located on chromosome O1 and two on O5 (**Figure 1**). Five QTL were identified for *S*. Both *S* QTL on O6 co-localized with *T*_0_ QTL, while one of them was also found to co-localize with an LAR QTL. The third QTL for *S* on O9 was located close to a LAR QTL. Genetic positions, LOD scores, and additive effects of all QTL are summarized in **Table 4**. LAR QTL on O9 and O5 revealed highest LOD scores of 7.04 and 6.62, respectively. LOD scores of the two QTL for *S* on O6 were found to be highest with 8.0 and 4.6.

**Table 4:** List of QTL for leaf appearance rate (LAR), slope (*S*), and intercept (*T*_0_) including confidence intervals, LOD-scores, % variation explained (*R^2^*) and effects of the P1 allele.

Trait	Chromosome	Position (cM)	Confidence interval (cM)	Nearest marker	LOD-score	*R^2^* (%)	Additive effect
LAR	1	24	22–26	S1066	4.68	12.5	-0.504
	1	58	54–62	S0528	6.62	17.2	0.795
	1	78	72–80	S0598	2.70	7.5	-0.508
	3	32	28–34	S0623	3.42	9.3	-0.388
	5	0	0–2	S0359	2.70	7.6	-0.425
	5	20	18–28	S0975	4.61	12.4	0.541
	6	32	30–36	S0509	2.99	8.2	0.593
	7	28	22–34	S0289	4.09	11.0	0.504
	7	80	64–84	S0728	2.96	8.1	-0.398
	9	38	24–48	S0501	7.04	18.3	0.623
*S*	1	74	68–80	S1119	4.12	11.7	-4.972
	4	114	108–116	S1115	4.42	12.4	-5.217
	6	12	46–18	S1114	8.00	21.3	-8.885
	6	32	30–36	S0509	4.55	12.7	-6.891
	9	48	44–52	S0501	3.98	11.2	-4.797
*T*_0_	1	74	66–80	S1119	2.91	8.5	64.62
	6	12	4–20	S1114	4.49	12.6	100.54


**FIGURE 2:**
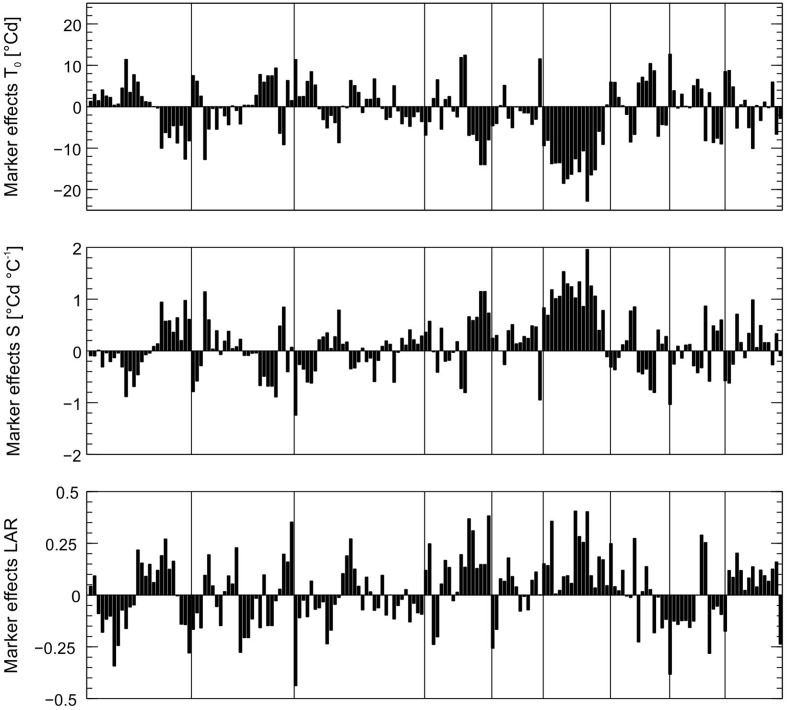
Effects of 176 SNP markers for the parameters leaf appearance rate (LAR, 1/°Cd), slope (*S*, in °Cd/°C) and intercept (*T*_0_, in °C) estimated by genome-wide analysis. Markers are shown in the order of their position on the genome, vertical lines indicate linkage groups.

Genome-wide marker effects for the model parameters LAR, *S*, and *T*_0_ were estimated by rrBLUP and are displayed in **Figure 2**. Large marker effects on O6 support linkage-mapping results, which indicated a promising hotspot region on O6.

### Genome-Based Prediction of Curding Time

**FIGURE 3:**
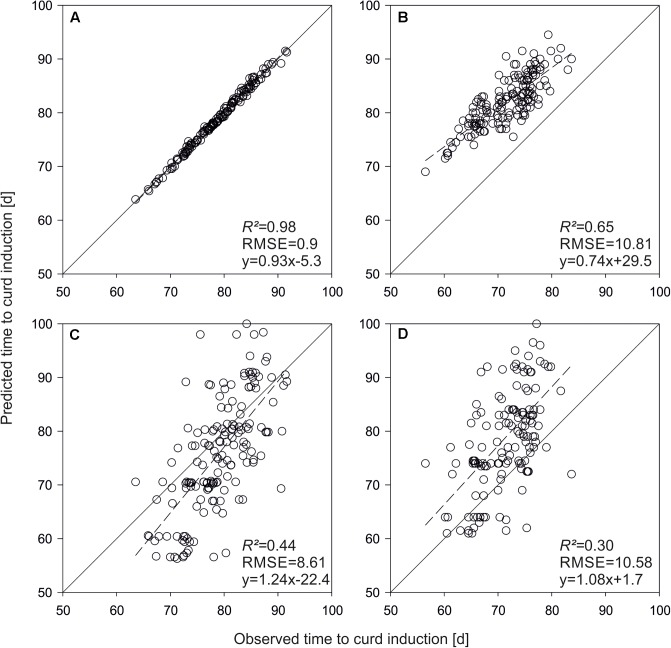
Predicted vs. observed mean time to curd induction of the DH lines used for model parameterization. Shown are means of seven greenhouse trials **(A,C)** and means of field trials in Zeewolde 2011 and 2012 **(B,D)**. Predictions are based on original parameters **(A,B)** and QTL effects **(C,D)**.

To get an idea about the power of the phenological model, curd induction of the parameterization set was simulated with both QTL-effects and original parameters. A very high prediction accuracy of *R^2^* = 0.98 was observed applying original parameters to predict time to curd induction of the parameterization trials. **Figures 3A,C** show means across seven temperature treatments. *R^2^* decreased to 0.44 if original parameters were replaced by QTL-based estimates. A similar situation with an overall reduced *R^2^* was observed if mean time to curd induction of field data from Zeewolde 2011 and 2012 was simulated (**Figures 3B,C**). *R^2^* diminished to 0.47 if mean curd induction of F1 hybrids derived from the parameterization set in field trials in Ócsa 2011, 2012 and Zeewolde 2012 was modeled using original parameters (data not shown).

**FIGURE 4:**
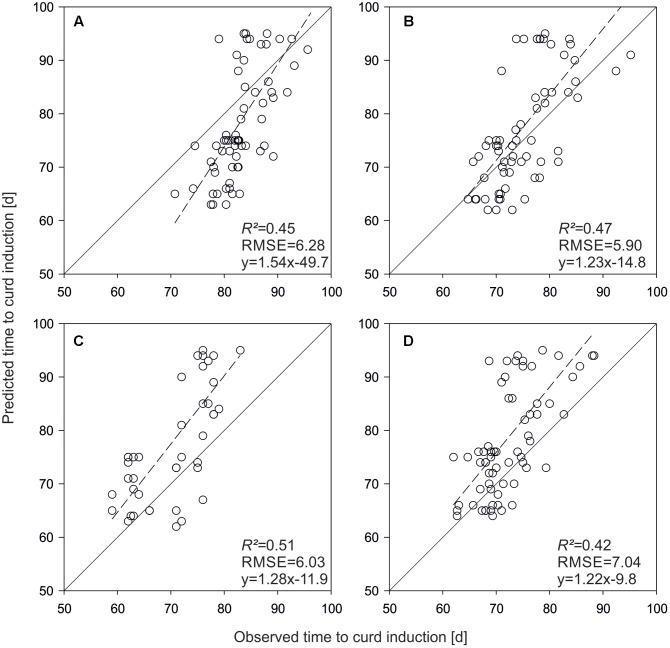
Predicted vs. observed time to curd induction of the validation set in field trials conducted in Rostock 2013 **(A)**, Ruthe 2013 **(B)**, Zeewolde 2011 **(C)**, and Zeewolde 2012 **(D)**. All predictions are based on QTL effects.

For model evaluation, QTL effects were used as parameter inputs to predict time to curd induction of the validation set. **Figure 4** shows predicted vs. observed time to curd induction of different field trials. In Rostock, mean observed time to curd induction was 83.5 days (±11.5), while predicted time to curd induction was 79.1 days (±4.9). The coefficient of determination was *R^2^* = 0.45 (**Figure 4A**). With *R^2^* = 0.47, prediction accuracy was slightly higher in Ruthe (**Figure 4B**), while the model performed best in the Zeewolde trial in 2011 (*R^2^* = 0.51, **Figure 4C**). Interestingly, prediction of independent lines resulted in higher *R^2^* values if compared to the parameterization set. However, the slope was 1.08 for the parameterization set (**Figure 3D**) but ranged between 1.22 and 1.54 when curding time of independent lines was modeled (**Figure 4**).

**FIGURE 5:**
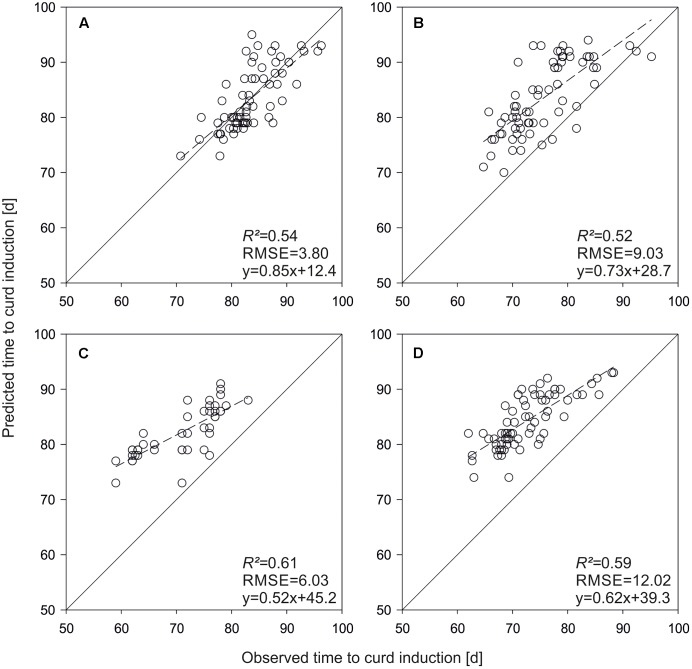
Predicted vs. observed time to curd induction of the validation set in field trials conducted in Rostock 2013 **(A)**, Ruthe 2013 **(B)**, Zeewolde 2011 **(C)**, and Zeewolde 2012 **(D)**. All predictions are based on genomic selection.

As an alternative method, genome-wide marker effects were estimated. The GS model led to higher prediction accuracies in all field trials if applied on the validation set (**Figure 5**). *R^2^* increased on average by 10.3%. However, a relatively strong bias was observed in the Zeewolde trials (**Figures 5C,D**).

**FIGURE 6:**
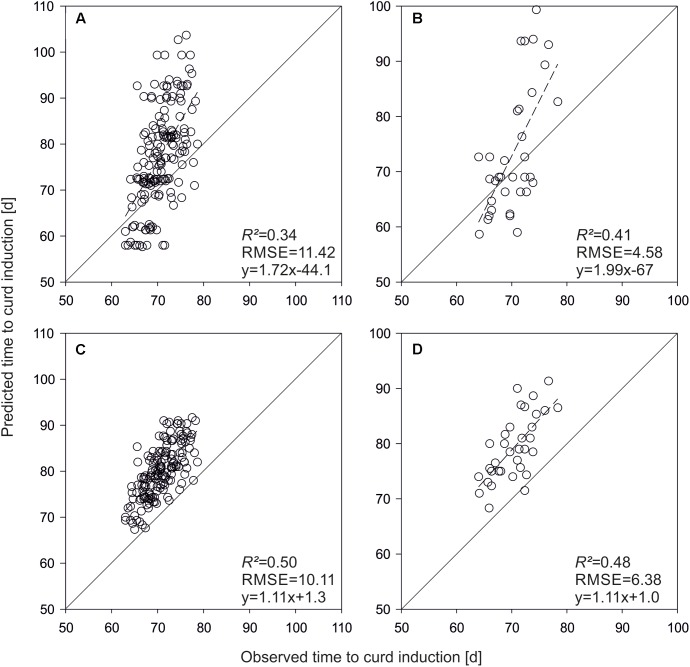
Predicted vs. observed time to curd induction of F1-hybrids derived from lines used for model parameterization **(A,C)** and the independent validation set **(B,D)**. Shown are means of three field trials in Ócsa 2011 and Zeewolde 2011 and 2012. Predictions are based on QTL effects **(A,B)** and genomic selection **(C,D)**.

Applying the QTL-based model on test hybrids developed from the parameterization set resulted in moderate prediction accuracies (*R^2^* = 0.34, **Figure 6A**). If QTL effects were replaced by genome-wide marker effects, *R^2^* increased to 0.50 (**Figure 6C**). The QTL based simulation of time to curd induction of F1-hybrids derived from the validation set revealed also moderately good results (*R^2^* = 0.41, **Figure 6B**), which were outperformed by the GS based model (*R^2^* = 0.48; **Figure 6D**).

## Discussion

### QTL Analyses and Genomic Selection

QTL were found for all examined parameters. QTL for *S* and *T*_0_ were almost congruent in their positions, which was not surprising, as *S* and *T*_0_ were highly correlated (*r* = -0.95) and lines with a steep slope do have a low intercept and *vice versa*. Similar to the present study, Uptmoor et al. (2008) found QTL for ‘slope’ on chromosomes O9 and O6 in a *B. oleracea* var. *alboglabra* × *B. oleracea* var. *italica* cross. QTL for ‘slope’ were not detected on chromosomes O4 or O1 but an additional QTL was identified on O3, which was not confirmed in the present study. Uptmoor et al. (2008) detected four QTL for the parameter ‘intercept.’ One was also located O6 but three further QTL on O3, O4, and O5 were not verified in this study. Even if positions are not completely comparable since both studies used different markers and linkage maps, clear parallels between the detected QTL are evident.

At the positions of three of the five QTL for slope, across environment QTL with significant QTL × environment interactions were detected in an earlier study using the same population and phenotype data (Hasan et al., 2016), indicating that input parameters of ecophysiological models are suitable traits for QTL detection. In contrast to the statistical interaction of a QTL with its environments (i.e., different effect sizes in different environments), model parameters display the trait’s response to an environmental factor allowing precise estimations of a new genotype in untested environments (Onogi et al., 2016). The QTL regions on O6 harbor *BoAP1-c* and *BoAP1-a*, the latter of which was suggested to interact with temperature (Labate et al., 2006). The QTL region on O9 harbors an *FLC* paralog and a *FRIGIDA LIKE* (*FRL*) ortholog (Hasan et al., 2016).

Usually, a relatively large sample size is required for QTL analyses and several hundreds of lines are recommended for good results (Hyne et al., 1995; Beavis, 1998; Lynch and Walsh, 1998). In general, the higher the number of genotypes used for analyses, the more accurate are the results. QTL with relatively small effects, so called minor QTL, are detected only in large populations, while major QTL can still be found with fewer genotypes (Vales et al., 2005; Slate, 2005), which, however, leads to an underestimation of the total number of QTL, while QTL effects are overestimated since a part of the variation is due to undiscovered minor QTL (Beavis, 1994; Xu, 2003). All significant QTL were tested for QTLxQTL interactions by pairwise linear regression analysis. The only significant interaction was detected for the parameter slope (QTL at 114 cM on O4 and at 12 cM on O6). However, including the interaction effect into the model improved estimations for the slope only slightly and had no positive impact on the simulation studies (data not shown). Hill et al. (2008) found that additive variance accounts often for close to 100% of the total genetic variance, which might be the reason for the missing positive impact of QTLxQTL interactions on the QTL-based simulation model of the present study.

Genomic selection is a useful tool to overcome these limitations as effects are assigned to all markers. GS was used as an alternative method for model parameterization and regions with large marker effects on O4 and O6 reflect findings from linkage mapping quite well; but in contrast to the QTL model, GS still accounts for genome regions with minor effects.

### Genome-Based Simulation Models

The original model was able to predict time to curd induction by means of chosen parameters in a satisfactory manner. The possibility to simulate cauliflower development from temperature data only has already been shown (Olesen and Grevsen, 2000). The QTL-based approach was rather successful as well, although predictions based on original parameters were more precise. Assumptions made by Reymond et al. (2003) and Uptmoor et al. (2008) that replacement of model parameters by QTL effects would lead to higher prediction accuracies due to the reduction of random errors were not confirmed in the present study. While the prediction of hybrids derived from the DH population was possible, prediction accuracies were not as high as those of DH lines *per se*.

Considering simulation studies carried out on independent genotypes of the cross, results adequately reflected expectations if compared to similar studies without independent genotypes (Quilot et al., 2005; Yin et al., 2005a; White et al., 2008; Uptmoor et al., 2012). However, Bogard et al. (2014) attained relatively high prediction accuracies (mean *R^2^* = 0.58) for predicting heading date of independent bread wheat genotypes using a common phenology model and genome-wide association studies for QTL detection. Less precise predictions of the present study may result from existing variation in juvenile-to-adult transition, for which the curd-induction model did not account.

Unexpectedly, the genome-based prediction of independent DH lines exceeded the accuracy of the simulations of lines used for parameterization and even the prediction of F1 hybrids resulted in a slightly higher *R^2^.* If subsamples of a population are large enough, different random subsamples will provide similar results regarding QTL positions and effects, hence, prediction accuracies for independent lines are likely to be on the same level as for lines used as parameterization set. Therefore, higher accuracies for independent lines in this study can only be explained by chance. In fact only *R^2^* values were higher, while slopes > 1 illustrate that the variability in curding time within the validation set was overestimated.

Simulations using genome-wide marker effects led to more precise predictions in comparison to QTL-based simulations, which held true for both independent DH lines of the validation set and test hybrids. The major reason might be explained by missing effects of minor loci in the QTL model. Comparing different models to predict hybrid performance in maize, Guo et al. (2013) found the genome-wide prediction approach being more accurate than predictions based on QTL effects.

In the present study, a simple approach with only three model parameters was used to predict time to curd induction of cauliflower lines and hybrids. Combining GS and ecophysiological models could reduce efforts spent in field trials to test germplasm across wide ranges of different environments *in silico* since GS models are able to predict phenotypes only in relatively similar environments while integrated models account for both genotypic and environmental variations in equal measure. Even though model parameterization requires large phenotyping efforts under controlled conditions, more precise predictions may be attained from more complex models.

The uncertainty about juvenile-to-adult transition might be considered as main error source impairing prediction accuracies. Juvenility is assumed to be independent from temperature and to end after a certain number of leaves are initiated (Salter and James, 1974; Hand and Atherton, 1987). Since large variations in juvenile-to-adult transition are present in cauliflower cultivars (Booij and Struik, 1990), certain variation may exist in the examined population as well. Genotype specific estimations of leaf numbers at the end of juvenility should be made in further experiments via reciprocal transfers to improve the model’s accuracy by integrating the onset of sensitivity to temperature.

In the present model, LAR was assumed to be constant throughout the vegetative phase. However, it is known that LAR changes with time and more sophisticated models already took this into account (Kage and Stützel, 1999). Similar approaches are conceivable to further improve simulation accuracies of integrated modeling approaches.

While the model of the present study simulates the development of cauliflower until visible curd induction, other phenology models already implemented curd development to predict harvest time (Pearson et al., 1994; Olesen and Grevsen, 2000). The practical use of a model predicting harvest time instead of time of curd induction is undoubtedly higher since accurate harvest-time predictabilities in diverse environments are a great challenge not only for growers but also for breeders.

## Conclusion

We conclude from the present study that parameters describing the response of a genotype to an environmental factor are suitable traits for QTL detection. Identified loci reflect across environment QTL with significant QTL × environment interactions, while having beneficial effects since the response to the influencing factor is quantifiable. The latter allows predicting untested genotypes in a broad range of untested environments. Simulation studies carried out on independent genotypes comprising new DH lines and hybrids in new environments, revealed that models integrating GS with ecophysiological modeling provides better results than combined QTL and ecophysiological models do since GS based approaches take minor effect loci into account.

## Author Contributions

The trials were carried out by AR, YH, and WB. Experiments were designed by RU and the data was analyzed by AR and YH. The manuscript was written by AR, revised by RU and approved by WB.

## Conflict of Interest Statement

The authors declare that the research was conducted in the absence of any commercial or financial relationships that could be construed as a potential conflict of interest.
